# Neutrophil-to-lymphocyte ratio associated with symptomatic saccular unruptured intracranial aneurysm

**DOI:** 10.1186/s40001-023-01608-3

**Published:** 2024-01-11

**Authors:** De-Xiang Zheng, Yi-Yang Lv, Xiao-Jing Zhang, Jie-Shun Ye, Jian-Xing Zhang, Cha Chen, Bin Luo, Dan Yan

**Affiliations:** 1 Department of Clinical Laboratory, Guangdong Provincial Hospital of Chinese Medicine, Guangzhou, China; 2 The Second Clinical College of Guangzhou University of Chinese Medicine, Guangzhou, China; 3 Department of Blood Transfusion, Nanfang Hospital, Southern Medical University, Guangzhou, China; 4 Department of Epilepsy Center, The Second Affiliated Hospital, Guangzhou University of Chinese Medicine, Guangzhou, China; 5 School of Civil Engineering and Transportation, South China University of Technology, Guangzhou, 510640 China; 6 Department of Neurosurgery, The Eighth Affiliated Hospital, Sun Yat-Sen University, Shenzhen, 518033 China; 7 Department of Ultrasound, The Second Affiliated Hospital, Guangzhou University of Chinese Medicine, Guangzhou, China

**Keywords:** Unruptured intracranial aneurysm, Symptomatic, Inflammation, Neutrophil-to-lymphocyte ratio, Lymphocyte-to-monocyte ratio

## Abstract

**Background and purpose:**

Whether symptomatic unruptured intracranial aneurysms (UIAs) lead to change in circulating inflammation remains unclear. This study aims to evaluate the role of hematological inflammatory indicators in predicting symptomatic UIA.

**Methods:**

Adult patients diagnosed with saccular intracranial aneurysm from March 2019 to September 2023 were recruited retrospectively. Clinical and laboratory data, including the white blood cells (WBC), neutral counts (NEUT), lymphocyte counts (LYM), and monocyte counts (MONO) of each patient, were collected. The neutrophil-to-lymphocyte ratio (NLR) and lymphocyte-to-monocyte ratio (LMR) were calculated as NLR = NEUT/LYM, LMR = LYM/MONO, SII = PLT*NEUT/LYM. The hematological inflammatory indicators were compared in symptomatic saccular and asymptomatic UIA patients. Multivariable logistic regression analyses were performed to explore the factors predicting symptomatic UIA.

**Results:**

One hundred and fifty UIA patients with a mean age of 58.5 ± 12.4 were included, of which 68% were females. The NLR and LMR were significantly associated with symptomatic UIA, and the association remained in small UIAs (< 7 mm). The multiple logistic regression analysis showed that NLR was independently associated with symptomatic UIA. On ROC curve analysis, the optimal cutoff value of NLR to differentiate symptomatic from asymptomatic was 2.38. In addition, LMR was significantly associated with symptomatic UIA smaller than 7 mm.

**Conclusion:**

There was a significant correlation between NLR and symptomatic UIA. The NLR was independently associated with symptomatic UIA.

## Introduction

Unruptured intracranial aneurysms (UIAs) are pathologically confined dilations of the intracranial artery walls with a population prevalence of approximately 3.2%. They are the main cause of spontaneous subarachnoid hemorrhage (SAH) [1]. Once an aneurysm ruptures, the disability and mortality rates are very high. Inflammation is thought to play an important role in the development, progression, and rupture of the intracranial aneurysm [2–5]. A large number of macrophages, white blood cells (WBCs), and inflammatory factors are observed in the ruptured intracranial aneurysm tissues and peripheral blood of aneurysmal SAH patients [6–8].

Previous studies have shown that the risk of rupture was significantly higher in symptomatic UIA patients than in asymptomatic patients [11]. In addition, the aneurysm wall enhancement (AWE) on high-resolution vessel wall imaging (HR-VWI) was significantly associated with symptomatic UIA [9–13]. As AWE is a manifestation of local inflammation [14–19], it is believed that the local inflammation level increases in symptomatic UIA. Moreover, it was reported that the neutrophil-to-lymphocyte ratio (NLR), a widely recognized circulating inflammation marker, was significantly associated with AWE of UIA [20]. However, the change in circulating inflammatory indicators in symptomatic UIA patients remains unclear. This study aimed to assess the correlation between the level of circulating inflammatory indicators and symptomatic UIAs.

## Materials and methods

### Study population

Eighty-eight consecutive patients with saccular UIAs were identified from a prospectively maintained database between March 2019 and September 2023 at the department of Neurosurgery, Guangdong Provincial Hospital of Traditional Chinese Medicine, and Nanfang Hospital of Southern Medical University. The study protocols were approved by the ethics committees of Guangdong Provincial Hospital of Traditional Chinese Medicine (IRB number: ZE2023-183-01).

The inclusion criteria were the diagnosis of IAs, age > 18 years, and saccular UIA identified on MR angiography or DSA. UIA patients who were regarded as symptomatic were as follows: acute headache (intense headache at onset with resolution in the following 72 h); chronic or recurrent headache (headache disappeared or with a marked improvement after surgical intervention of the aneurysm) [13, 21]; or cranial nerve palsy (such as the third, second, fourth, fifth and sixth nerve palsy) [22] caused by the unruptured intracranial aneurysms. Aneurysm status which was symptomatic or evolving during following up was defined as unstable UIAs. Patients with a known history of infection, stroke, heart disease, autoimmune disease, hematological diseases, cancer, and chronic liver and kidney insufficiency were excluded from this study.

The clinical data, including age, sex, hypertension, diabetes, current smoking, and drinking status, were recorded. Moreover, we acquired data, including the number, location, size, and morphology of aneurysms. PHASES score of every aneurysm was calculated.

### Blood examination

All patients underwent complete blood cell count analysis within 24 h after admission. The WBC, neutral counts (NEUT), lymphocyte counts (LYM), monocyte counts (MONO), platelet counts (PLT) of each patient were recorded. The NLR, Systemic Immune-Inflammation Index (SII), and lymphocyte-to-monocyte ratio (LMR) of each patient were calculated as follows: NLR = NEUT/LYM, LMR = LYM/MONO, SII = PLT* NEUT/LYM.

### Statistical analysis

We used SPSS 22.0 software for statistical analysis. Laboratory data, such as the NLR, LMR, SII, WBC, NEUT, LYM, and MONO, which obeying normal distribution were presented as mean ± SD and were compared using the student’s t-test. The clinical data such as sex, hypertension, diabetes, and current smoking were expressed as the number of cases and percentage. They were compared with the different groups using Fisher's exact or chi-square test. *P* < 0.05 was considered statistically significant. Multivariate logistic regression analyses were conducted to determine which factors were independent risk factors for symptomatic UIA after adjusting for variables with *p* < 0.1 in the univariate comparisons. The NLR value for identifying symptomatic from asymptomatic UIAs was analyzed using receiver operating characteristic (ROC) curve analysis.

## Results

### Clinical characteristics

During a 4-year study period, 226 patients with UIAs were retrospectively identified. After excluding patients for either defined criterion, 150 patients with saccular UIAs were included in this study, among them 85 located in ICA, 20 located in MCA, 45 located in posterior circulation. Seventy-six patients were excluded: 28 patients with fusiform aneurysms, 8 patients with a known history of infection, 21 patients with stroke, 12 patients with heart disease, 6 patients with cancer, and 1 patient with kidney insufficiency were excluded from this study. There were 54 symptomatic patients, among them, 9 had cranial nerve palsy, 28 had acute headache, 12 had chronic headache, 5 had recurrent headache. The characteristics of the patients and aneurysms are shown in Table 1.

**Table 1:** Characteristics of aneurysm and laboratory parameter in symptomatic and asymptomatic UIA patients

	Symptomatic (*n* = 54)	Asymptomatic (*n* = 96)	*p* value
Age (yr)	56.7 ± 14.2	59.3 ± 11.1	0.231
Female	36 (66.7%)	66 (68.8%)	0.793
Hypertension	22 (40.7%)	42 (43.8%)	0.721
Diabetes	5 (9.3%)	8 (8.3%)	0.847
Smoking	9 (16.7%)	16 (16.7%)	1.000
Size (mm)	6.29 ± 5.15	4.56 ± 2.65	0.024
Location			0.703
Anterior circulation	40	65	
Posterior circulation	14	31	
WBC (× 10^9^/L)	6.74 ± 2.56	6.38 ± 1.88	0.334
LYM (× 10^9^/L)	1.87 ± 0.58	2.04 ± 0.58	0.092
NEUT (× 10^9^/L)	4.22 ± 2.38	3.67 ± 1.49	0.130
MONO (× 10^9^/L)	0.40	0.41	0.955
NLR	2.60 ± 2.00	1.89 ± 0.84	0.017
LMR	4.35 ± 1.70	5.07 ± 2.38	0.050
SII	485.31	416.78	0.115
PHASES score	3.37 ± 2.84	2.66 ± 2.48	0.110

*NLR* neutrophil-to-lymphocyte ratio, *LMR* lymphocyte-to-monocyte ratio, *SII* Systemic Immune-Inflammation Index

### NLR was independently associated with symptomatic UIA

The aneurysm size was larger in the symptomatic UIA group than in the asymptomatic group (6.29 mm vs. 4.56 mm, *p* = 0.024). Symptomatic UIA patients were more likely to have higher levels of NLR (*p* = 0.017) and low levels of LMR and LYM (*p* = 0.050, *p* = 0.092, respectively) than the asymptomatic patients (Table 1). These variables (*p* ≤ 0.1) were subsequently entered into a multiple logistic regression model to determine the risk factors for symptomatic UIA. The results showed that in these variables the NLR (OR: 1.468, 95% CI 1.092–1.975, *p* = 0.011) and size (OR:1.125, 95% CI 1.020–1.241, *p* = 0.018) were independently associated with symptomatic UIA (Table 2).

**Table 2:** Multiple logistic regression analysis for symptomatic UIA

	OR	95% CI	*P* value
Size	1.125	1.020–1.241	0.018
LYM	1.081	0.537–2.174	0.828
LMR	0.973	0.791–1.196	0.793
NLR	1.468	1.092–1.975	0.011

*NLR* neutrophil-to-lymphocyte ratio, *LMR* lymphocyte-to-monocyte ratio

**Fig. 1:**
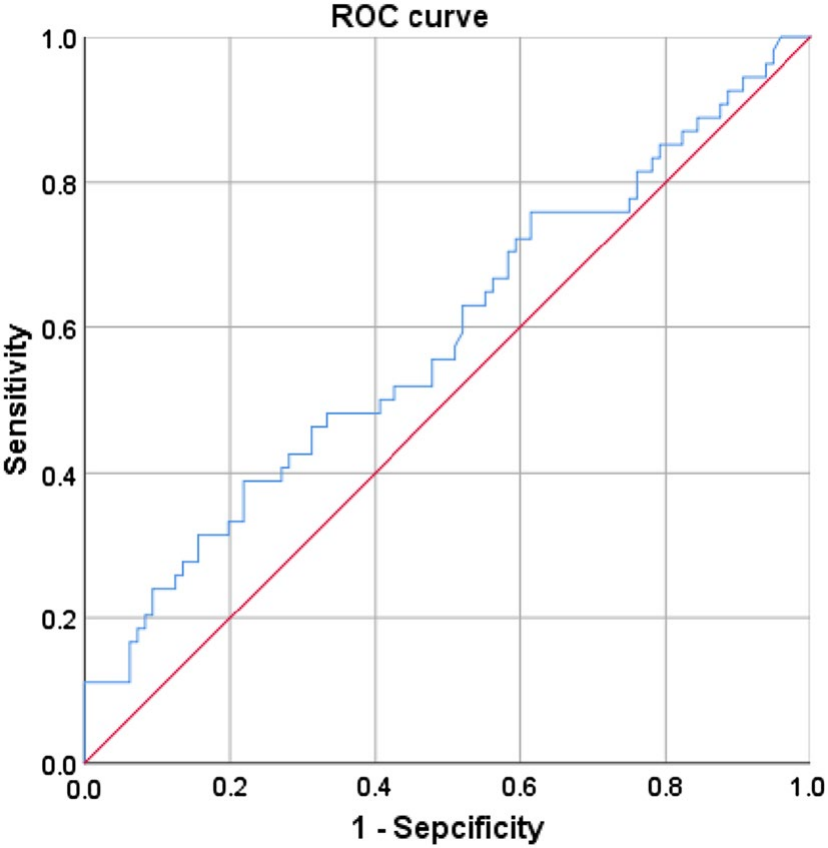
Receiver operating characteristic curve of NLR to differentiate symptomatic UIA, the area under the curve was 0.584, the cutoff value of NLR was 2.38

Furthermore, the optimal cutoff value of NLR to differentiate symptomatic UIA from asymptomatic UIA was 2.38 on ROC curve analysis, and the area under the curve was 0.584 (Fig. 1). Using a cutoff value of 2.38 for NLR, the sensitivity and specificity were 0.389 and 0.781, respectively. In the subgroup with saccular UIAs smaller than 7 mm, the LMR (OR:0.759, 95% CI 0.609–0.947, *p* = 0.014) was independently associated with symptomatic UIAs in these variables (Tables 3 and 4).

**Table 3:** Characteristics of aneurysm and laboratory parameter in symptomatic and asymptomatic patients of saccular UIA smaller than 7 mm

	Symptomatic (*n* = 38)	Asymptomatic (*n* = 79)	*p* value
Age (yr)	55.7 ± 15.2	59.1 ± 10.2	0.222
Female	24 (63.2%)	54 (68.4%)	0.577
Hypertension	18 (47.4%)	37 (46.8%)	0.957
Diabetes	4 (10.5%)	7 (8.9%)	0.746
Smoking	7 (18.4%)	13 (16.5%)	0.791
Size (mm)	3.88 ± 1.47	3.58 ± 1.44	0.286
Location			0.561
Anterior circulation	26	54	
Posterior circulation	12	25	
WBC (× 109/L)	6.93 ± 2.83	6.53 ± 1.87	0.358
LYM (× 10^9^/L)	1.84 ± 0.55	2.12 ± 0.58	0.115
NEUT (× 10^9^/L)	4.30 ± 2.59	3.74 ± 1.46	0.215
MONO (× 10^9^/L)	0.40	0.41	0.256
NLR	2.46 ± 1.76	1.84 ± 0.76	0.042
LMR	4.31 ± 1.73	5.44 ± 2.40	0.011
SII	379.94	422.72	0.409
PHASES	2.0	1.0	0.561

*NLR* neutrophil-to-lymphocyte ratio, *LMR* lymphocyte-to-monocyte ratio, *SII* Systemic Immune-Inflammation Index

**Table 4:** Multiple logistic regression analysis for symptomatic patients with saccular UIA smaller than 7 mm

	OR	95% CI	*p* value
LMR	0.759	0.609–0.947	0.014
NLR	1.296	0.867–1.938	0.488

*NLR* neutrophil-to-lymphocyte ratio, *LMR* lymphocyte-to-monocyte ratio

## Discussion

To the best of our knowledge, this is the first study investigating the relationship of circulating inflammatory indicators between symptomatic saccular UIA and asymptomatic UIA. Our data confirmed that in saccular UIA patients, the elevation of the baseline NLR was associated with symptomatic and an independent risk factor for symptomatic saccular UIA. On ROC curve analysis, the optimal cutoff value of NLR to differentiate symptomatic UIAs from asymptomatic was 2.38. Moreover, in saccular UIAs smaller than 7 mm, the LMR was the only independent risk factor of symptomatic UIA.

Recently, neuroinflammation has drawn increasing attention, and numerous studies have confirmed that inflammation play critical roles in the pathogenesis and progression of UIAs [2, 3]. Lymphocytes, neutrophils, and monocytes are traditional inflammatory cells, and it was reported that lymphocytes have neuroprotective effects and could improve neurological function [23]. Neutrophils played a key role in the inflammatory mechanisms seen in sarcopenia, which was independently associated with the mRS score at 6 months of patients with subarachnoid hemorrhage treated by endovascular coiling [24]. NLR, LMR and SII, which provide a simple way to assess the inflammatory status, are novel and inexpensive inflammation markers that have been widely used as inflammation indicators in recent years. It was showed that the elevated NLR and SII levels, and the decreased LMR level were more common in acute and chronic inflammatory diseases, immune diseases and cancers. Moreover, these conditions were also observed in acute coronary heart disease [25], heart failure [26], and acute ischemic brain stroke [27, 28]. In addition, several previous literatures reported that NLR, LMR and SII were crucial blood inflammatory indicators in aneurysmal SAH [29–31]. Previous studies on NLR, LMR and SII in intracranial aneurysms were inadequate, especially in UIA.

Until now, no studies have reported the clinical value of NLR in symptomatic UIAs. The present study showed that elevation of the baseline NLR was associated with symptomatic saccular UIA. Moreover, NLR was an independent risk factor for symptomatic UIA, indicating an increased level of inflammation in symptomatic UIA patients than in asymptomatic patients. A previous literature reported that high level of NLR showed significant relationship with the size of UIAs, and an elevated NLR may be a clinical feature before the aneurysm rupture [32]. We also showed that aneurysm size was an independent risk factor of symptomatic UIA, indicating that it was unstable, similar to previous studies.

Moreover, we also showed that the optimal cutoff value of NLR to differentiate symptomatic UIA from asymptomatic was 2.38, indicating that NLR ≥ 2.38 might identify unstable saccular UIAs. In addition, we also found that LMR was significantly associated with symptomatic UIAs smaller than 7 mm, indicating that circulating inflammation of patients with small aneurysm might also change.

However, this study has several limitations. First, it was a retrospective study with case selection bias. Second, the number of patients in this study was relatively small, and the results showed that not all inflammatory indicators were significantly higher in the symptomatic UIA group compared to the asymptomatic group, in addition, we did not test and analyze CRP or other inflammatory markers in patients at admission. Thirdly, although we excluded definite infection at admission, our assessment of infection may have been inadequate. Fourth, peripheral blood NLR, LMR and SII may be affected by concomitant diseases, such as hypertension and diabetes mellitus, which might have biased the results. However, this study included NLR in the multifactorial regression analysis, which was found to be an independent risk factor, so the bias may be small. Fifth, inflammatory features in peripheral blood do not directly reflect inflammatory infiltration of the IA wall, we intend to further elucidate its significance in conjunction with pathological studies. Finally, no follow-up study was performed to clarify that UIA with high baseline NLR had a higher rate of aneurysm progression. A follow-up study is needed for confirmation.

## Conclusions

NLR was an independent predictor of symptomatic UIA, and its increase may correlate with a high rupture risk of saccular UIA. Symptomatic UIA patients showed high levels of inflammatory indicators, which provided certain hematology evidence that inflammation plays an important role in the formation and progression of UIAs.

## Data Availability

The data sets in this study are available from the corresponding author on reasonable request.

## Abbreviations

UIA: Unruptured intracranial aneurysms
SAH: Spontaneous subarachnoid hemorrhage
AWE: Aneurysm wall enhancement
HR-VWI: High-resolution vessel wall imaging
WBC: White blood cell
NEUT: Neutral counts
LYM: Lymphocyte counts
MONO: Monocyte counts
PLT: Platelet counts
SII: Systemic immune-inflammation index
NLR: Neutrophil-to-lymphocyte ratio
LMR: Lymphocyte-to-monocyte ratio
ROC: Receiver operating characteristic
